# Voluntary vitamin D testing: a decade-long study of utilisation patterns and impact on deficiency outcomes in Taiwan

**DOI:** 10.1017/S1368980025101183

**Published:** 2025-09-25

**Authors:** Hsiao-Yun Yeh, Mei-Lin Shih, Jei-Wen Chang, Liang-Yu Lin, Zih-Kai Kao, Ai-Ru Hsieh, Ping-Hsing Tsai, Jui-To Wang, Yu-Chun Chen

**Affiliations:** 1 Department of Medical Education, Taipei Veterans General Hospital, Taipei 112201, Taiwan; 2 Department of Family Medicine, Taipei Veterans General Hospital, Taipei 112201, Taiwan; 3 School of medicine, National Yang Ming Chiao Tung University, Taipei 112304, Taiwan; 4 Big Data Center, Taipei Veterans General Hospital, Taipei 112201, Taiwan; 5 Department of Pediatrics, Taipei Veterans General Hospital, Taipei 112201, Taiwan; 6 Division of Endocrinology and Metabolism, Department of Medicine, Taipei Veterans General Hospital, Taipei 112201, Taiwan; 7 Department of Information Management, Taipei Veterans General Hospital, Taipei 112201, Taiwan; 8 Department of Statistics, National Taipei University, New Taipei City 23741, Taiwan; 9 Department of Medical Research, Taipei Veterans General Hospital, Taipei 112201, Taiwan; 10 Department of Neurosurgery, Neurological Institute, Taipei Veterans General Hospital, Taipei 112201, Taiwan; 11 Department of Family Medicine, Taipei Veterans General Hospital Yuli Branch, Hualien 981002, Taiwan

**Keywords:** Vitamin D deficiency, Voluntary testing, Nutritional epidemiology, Nutritional status assessment, Public health nutrition

## Abstract

**Objective:**

Although guidelines recommend targeted vitamin D testing for high-risk populations, testing has increased globally. Limited studies have examined real-world testing patterns and their relationship with deficiency outcomes. This study investigates trends, demographic determinants and deficiency outcomes associated with voluntary vitamin D testing among Taiwanese adults.

**Design:**

A retrospective cohort study analysing electronic medical records to assess vitamin D testing trends, demographic predictors of deficiency and status changes following consecutive tests within 2 years. Vitamin D status was classified based on serum 25-hydroxyvitamin D levels as deficient (< 20 ng/ml), insufficient (20–29·9 ng/ml) or sufficient (≥ 30 ng/ml).

**Setting:**

A tertiary medical centre in Taiwan.

**Participants:**

Between 2013 and 2022, 13 381 outpatients underwent voluntary vitamin D testing. After excluding those aged < 18 years, with advanced renal disease, osteomalacia, rickets or hyperparathyroidism, 8383 were included in the final analysis.

**Results:**

Testing increased sharply after 2019. Although women underwent twice as many tests, men had a higher deficiency prevalence (56·94 % *v*. 53·01 %). Adults aged 18–34 years had the highest prevalence (67·81 %). Obstetrics and Gynecology specialists ordered the most tests, particularly for female infertility, with 65·73 % of patients deficient. Among those with repeat tests, deficiency prevalence decreased from 59.32 % to 43·25 %.

**Conclusions:**

The increase in voluntary vitamin D testing with demographic disparities highlights the importance of understanding testing behaviours and public health implications. Improved vitamin D status at follow-up suggests potential benefits in identifying high-risk individuals and emphasises the need for further research to evaluate outcomes and guide prevention strategies.

Vitamin D is crucial for maintaining bone health and regulating Ca and phosphate metabolism^(1,2)^. Sufficient levels of vitamin D have also been associated with a reduction in the risk of various acute and chronic diseases^(3–6)^. Despite its importance, vitamin D deficiency remains a global health challenge^(7–9)^, affecting populations across various geographic regions, age groups and socio-economic statuses^(10,11)^.

In response to the high prevalence of vitamin D deficiency, international health organisations have issued guidelines emphasising preventive strategies, such as lifestyle modifications to enhance vitamin D levels through increased sunlight exposure and dietary measures, including the intake of oily fish, eggs, vitamin D-fortified foods and supplements^(12–14)^.

These guidelines generally recommend vitamin D testing for high-risk groups (e.g. older adults, those with limited sun exposure, darker skin, malabsorption, chronic kidney disease, obesity or endocrine disorders), rather than advocating for widespread testing among the general population, to limit unnecessary testing and reduce healthcare costs^(15,16)^.

However, in practice, there has been a noticeable rise in vitamin D testing in many countries, including Australia, Canada and the UK^(17–19)^. This increase appears to stem from growing public awareness of vitamin D’s potential health benefits and the expanding body of research linking vitamin D deficiency to various diseases^(3–6)^. This discrepancy between guideline recommendations and real-world testing practices underscores the need to explore the factors associated with individuals’ seeking vitamin D testing, to get insights into their health behaviours and attitudes^(20,21)^.

In Taiwan, although the healthcare system provides near-universal coverage through the National Health Insurance^(22)^, vitamin D testing is not reimbursed and remains an out-of-pocket expense. At our centre, the cost of serum vitamin D testing is approximately 25–30 USD. This creates a unique scenario where patients’ health-seeking behaviours are shaped by their awareness and perceived benefits of vitamin D. This scenario provides an opportunity to study the health behaviours of individuals who actively choose to undergo vitamin D testing, offering insights into how demographic and clinical factors influence their decisions and how these behaviours impact their health outcomes.

Our study aims to investigate the trends and outcomes of voluntary vitamin D testing among ambulatory patients at a tertiary medical centre in Taiwan over a decade, from 2013 to 2022. By analysing the demographic characteristics associated with vitamin D deficiency and tracking changes in vitamin D status among those who underwent consecutive testing, we seek to provide insights into the practical impact of voluntary testing. These findings will help bridge the gap between clinical guidelines and real-world practices, offering information for shaping future public health policies and clinical interventions aimed at reducing vitamin D deficiency.

## Methods

### Study design and data source

**Figure 1.**
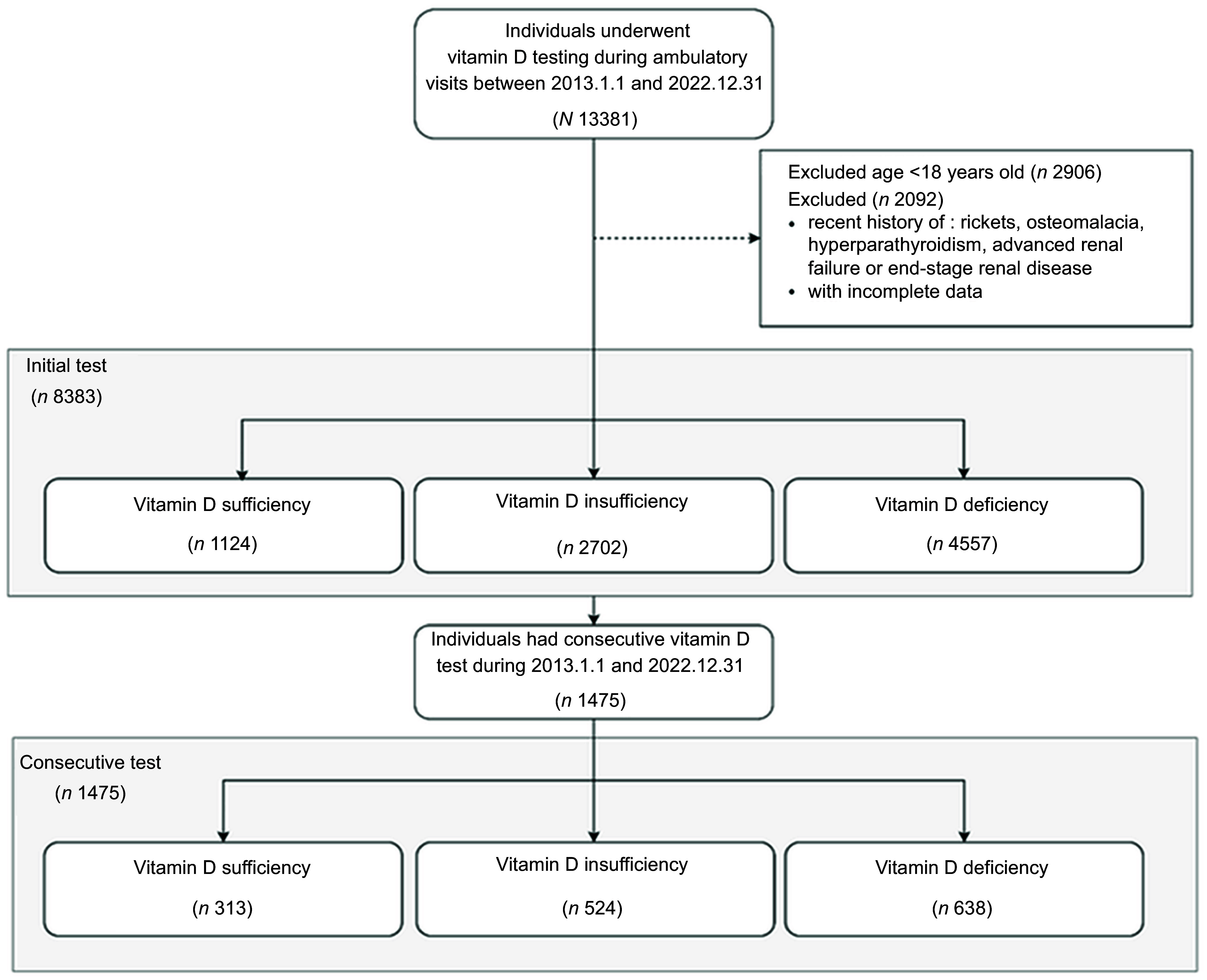
Study flow chart of individuals undergoing vitamin D testing (2013–2022). The flow chart illustrates the two-step exclusion process and final inclusion of 8383 participants based on age, clinical criteria and data completeness.

This retrospective cohort study investigated the trends in serum vitamin D testing utilisation among outpatients at a tertiary medical centre and factors associated with vitamin D deficiency prevalence from 2013 to 2022 (Figure 1). We also tracked the study population and analysed the patterns of changes in vitamin D status for those with consecutive vitamin D testing. All data were retrieved anonymously by the Medical Research Department. The study complied with the code of ethics of the World Medical Association (Declaration of Helsinki) and was approved by the International Review Board. Written informed consent was waived by the Ethical Review Committee due to the retrospective design of the study.

### Study cohort and data collection

#### Study cohort

The study included individuals aged 18 years or older who underwent vitamin D testing during ambulatory visits between 1 January 2013 and 31 December 2022 (Figure 1). The first vitamin D test during the study period was designated as the index test. To establish baseline health status and minimise confounding, only those with a documented visit history of at least 2 years prior to the index date were included. In this study, the term ‘voluntary vitamin D testing’ refers to physician-prescribed, non-reimbursed serum 25-hydroxyvitamin D tests initiated at the discretion of the physician and/or patient, outside of routine, insurance-covered practice. As these tests are not reimbursed by Taiwan’s National Health Insurance, they require out-of-pocket payment by patients. Individuals with advanced renal failure, end-stage renal disease, rickets, osteomalacia or hyperparathyroidism were excluded (see online supplementary material, Supplemental Table S1).

#### Utilisation patterns of initial vitamin D testing over study period

To assess trends in utilisation of vitamin D testing, the study period was divided into five two-year intervals: 2013–2014, 2015–2016, 2017–2018, 2019–2020 and 2021–2022. This allowed us to examine the distribution of initial vitamin D tests across these time periods.

#### Demographic information and data collection

Baseline demographic data were collected for each participant, including sex, age, BMI (BMI, calculated as kg/m^2^) and the specialty of the physician ordering the test. BMI categories were defined according to the Health Promotion Administration of Taiwan: underweight (BMI < 18·5), normal weight (18·5 ≤ BMI < 24), overweight (24 ≤ BMI < 27) and obese (BMI ≥ 27)^(23)^. For all analyses of deficiency-related factors, including demographic and clinical comparisons, the vitamin D status was based on each individual’s first recorded vitamin D test.

To evaluate how our tested cohort compares with the general population, we conducted a stratified comparison using data from the Nutrition and Health Survey in Taiwan (NAHSIT, 2017–2020), which employed multistage, probability-based sampling. We restricted our study sample to participants tested during the same period and stratified both datasets by sex and age group using NAHSIT categories (19–44, 45–64 and ≥ 65 years), comparing vitamin D levels and deficiency prevalence.

#### Diseases associated with initial vitamin D testing

Diseases associated with initial vitamin D testing were determined based on ICD-9-CM and ICD-10-CM diagnosis codes recorded during ambulatory visits within 1 year before or after the index date. If more than three diagnosis codes were recorded during a visit, the first three were considered. These codes were categorised into clinically meaningful groups using the Clinical Classifications Software developed by the Agency for Healthcare Research and Quality^(24)^, allowing us to identify disease clusters associated with outpatient vitamin D testing. To assess the temporal specificity of associated diagnoses, we conducted a sensitivity analysis limited to diagnoses recorded at the same ambulatory visit as the index vitamin D test (index visit). These diagnoses were categorised using the same Clinical Classifications Software and summarised separately in online supplementary material, Supplemental Table S4.

### Serum vitamin D assay and definition of vitamin D status

Serum 25-hydroxyvitamin D levels, considered the best marker for assessing vitamin D status^(25)^, were measured by electrochemiluminescence immunoassay. The mean and sd of 25-hydroxyvitamin D levels were calculated. Vitamin D status was defined as deficient (< 20 ng/ml), insufficient (20–29·9 ng/ml) or sufficient (≥ 30 ng/ml) based on established guidelines^(26,27)^. Participants were categorised accordingly, and vitamin D deficiency was analysed in relation to sex, age, BMI, medical specialty consulted for serum vitamin D testing and associated diseases or comorbidities.

### Consecutive vitamin D testing

Consecutive vitamin D testing was defined as any test performed at least 30 d after the initial test but within 2 years. The intervals between tests and changes in vitamin D status were recorded. A Sankey diagram was used to visualise changes in vitamin D status over time.

### Statistical analysis

Baseline descriptive variables were presented as percentages for categorical data. *χ* ^2^ tests were used to assess differences in the prevalence of vitamin D deficiency across categories such as sex, age group, BMI, medical specialty and comorbidities. We employed Z-tests to assess significant differences in vitamin D deficiency prevalence across each medical specialty and related disease, comparing the prevalence within each specialty and disease group to the overall study population. Multivariate logistic regression was used to calculate adjusted OR for sex, age and BMI, identifying factors associated with vitamin D deficiency. All analyses were performed using R version 4.3.2, with two-sided tests, and *P*-values < 0·05 considered statistically significant.

## Results

### Characteristics of the study population and utilisation trends of vitamin D testing

**Figure 2.**
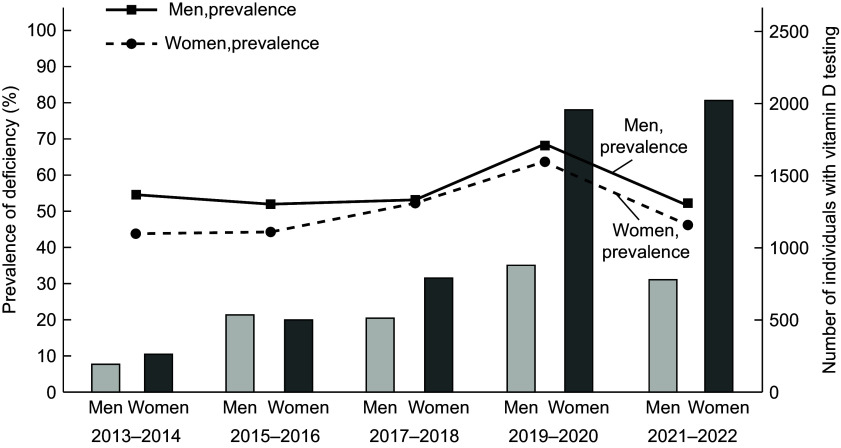
Trends in vitamin D deficiency prevalence and testing by year and sex (2013–2022). The left vertical axis represents the prevalence (%) of vitamin D deficiency among men and women, depicted as a line graph. The right vertical axis indicates the number of individuals undergoing vitamin D testing, displayed as a bar chart for men and women. The figure illustrates trends in vitamin D deficiency prevalence and the corresponding number of vitamin D testing conducted over different year periods, segmented by sex.

Between January 2013 and December 2022, 13 381 outpatients underwent 21 311 voluntary vitamin D tests. Among these, 8383 adult patients met the study’s inclusion criteria. Women accounted for nearly twice the proportion of vitamin D testing compared with men within the study population (65·62% *v*. 34·38%) (see online supplementary material, Supplemental Table S2). The number of tests increased significantly over the study period, especially after 2019, with more women being tested than men in all periods except 2015–2016 (Figure 2). Utilisation also rose across all age groups, with the highest testing rates in the 50–64 and 65–79 age groups, accounting for more than half of the total tests. Among the top medical specialties ordering vitamin D tests, Obstetrics and Gynecology (OB/GYN) had the highest proportion of consultations (1152; 13·74%), followed by endocrinology (890; 10·62%) and nephrology (558; 7·13%) (Table 1). Regarding comorbidities, 8·10% of tested individuals had essential hypertension, 5·73% had lipid disorders, and 5·36% had uncomplicated diabetes mellitus. Female infertility was also notably prevalent, affecting 5·20 % of the women (Table 1). In a sensitivity analysis using only index-visit diagnoses among vitamin D-deficient individuals, the most frequent categories included other endocrine disorders (*n* 475), thyroid disorders (*n*334), female infertility (*n*310), disorders of lipid metabolism (*n*227), and diabetes mellitus without complication (*n*219). Several of these categories – particularly endocrine disorders, thyroid conditions, lipid metabolism disorders, diabetes, and female infertility – were also among the most prevalent diagnoses in the one-year window analysis. This overlap supports the consistency of findings across timeframes, with particularly strong consistency observed in endocrine, metabolic and gynecologic conditions, including female infertility (see online supplementary material, Supplemental Table S4).

**Table 1.** Vitamin D status across various demographic and clinical categories in the study population (2013–2022)

Category	Total	Vitamin D status^*^						
		Deficiency		Insufficiency		Sufficiency		
	*n*	*n*	%	*n*	%	*n*	%	*P* value^†^
Overall	8383	4557	54·36	2702	32·23	1124	13·41	
Sex								< 0·001
Male	2882	1641	56·94	902	31·30	339	11·76	
Female	5501	2916	53·01	1800	32·72	785	14·27	
Age (years)								< 0·001
19–34	873	592	67·81	211	24·17	70	8·02	
35–49	1630	990	60·74	475	29·14	165	10·12	
50–64	2196	1132	51·55	720	32·79	344	15·66	
65–79	2397	1152	48·06	859	35·84	386	16·10	
≥ 80	1287	691	53·69	437	33·95	159	12·35	
BMI^‡^ (kg/m^2^) (429 missing values)								< 0·001
Underweight	862	495	57·42	257	29·81	110	12·76	
Normal	4099	2125	51·84	1347	32·86	627	15·29	
Overweight	1604	816	50·87	570	35·54	218	13·59	
Obese	1389	824	59·32	425	30·60	140	10·08	
Commonly associated diseases^§^								< 0·001
Essential hypertension	679	350	51·55	239	35·20	90	13·25	0·222
Disorders of lipid metabolism	480	223	46·46	180	37·50	77	16·04	0·002
Diabetes mellitus without complication	449	256	57·02	145	32·29	48	10·69	0·222
Endocrine disorders other than thyroid	378	235	62·67	117	31·20	23	6·13	0·002
Thyroid disorders	351	192	54·70	122	34·76	37	10·54	< 0·001
Osteoarthritis	372	163	43·82	140	37·63	69	18·55	0·220
Spinal and back disorders	330	160	48·48	115	34·85	55	16·67	0·228
Female infertility	286	188	65·73	83	29·02	15	5·24	< 0·001
Connective tissue disease	265	124	46·79	90	33·96	51	19·25	0·007
Chronic kidney disease	259	136	52·51	75	28·96	48	18·53	0·043
Coronary atherosclerosis and other heart disease	251	142	56·57	81	32·27	28	11·16	0·541
Osteoporosis	239	101	42·26	89	37·24	49	20·50	< 0·001
Medical specialties consulted								< 0·001
Obstetrics and Gynecology	1152	729	63·28	320	27·78	103	8·94	< 0·001
Endocrinology	890	429	48·20	331	37·19	130	14·61	< 0·001
Nephrology	598	322	53·85	204	34·11	72	12·04	0·534
Physical medicine and rehabilitation	581	246	42·34	226	38·90	109	18·76	< 0·001
Neurology	469	184	39·23	211	44·99	74	15·78	< 0·001
Gastroenterology and Hepatology	336	210	62·50	96	28·57	30	8·93	0·006
Infection	319	176	55·17	106	33·23	37	11·60	0·672
Family medicine	282	94	33·33	112	39·72	76	26·95	< 0·001
General surgery	275	151	54·91	76	27·64	48	17·45	0·056
Chest medicine	257	167	64·98	71	27·63	19	7·39	< 0·001

* Vitamin D status was defined as deficiency (< 20 ng/ml), insufficiency (20–29·9 /ml) and sufficiency (≥ 30 ng/ml).

† *P* values: Testing for differences in the prevalence of vitamin D deficiency between subgroups in each category, including sex, age groups, BMI categories, medical specialties, and commonly associated diseases, was conducted using chi-square tests.

The *P* values listed for each medical specialty or commonly associated disease represent statistical comparisons between specific groups and the overall study population using Z-tests.

‡ BMI categories are defined by the Taiwanese Ministry of Health and Welfare: Underweight (BMI ≤ 18·5 kg/m^2^), normal weight (18·5 ≤ BMI < 24 kg/m^2^), overweight (24 ≤ BMI < 27 kg/m^2^) and obese (BMI ≥ 27 kg/m^2^). (Ministry of Health and Welfare in Taiwan. Evidence-based guideline on adult obesity prevention and management. Available at https://www.hpa.gov.tw/Pages/EBook.aspx?nodeid=1788 Accessed on December 22, 2023).

§ Commonly associated diseases: Clinical Classifications Software (CCS) was used to categorise ICD-9-CM and ICD-10-CM diagnostic codes into clinically meaningful disease groups (CCS labels).

### Distribution of the serum vitamin D status and associated factors of vitamin D deficiency

**Figure 3.**
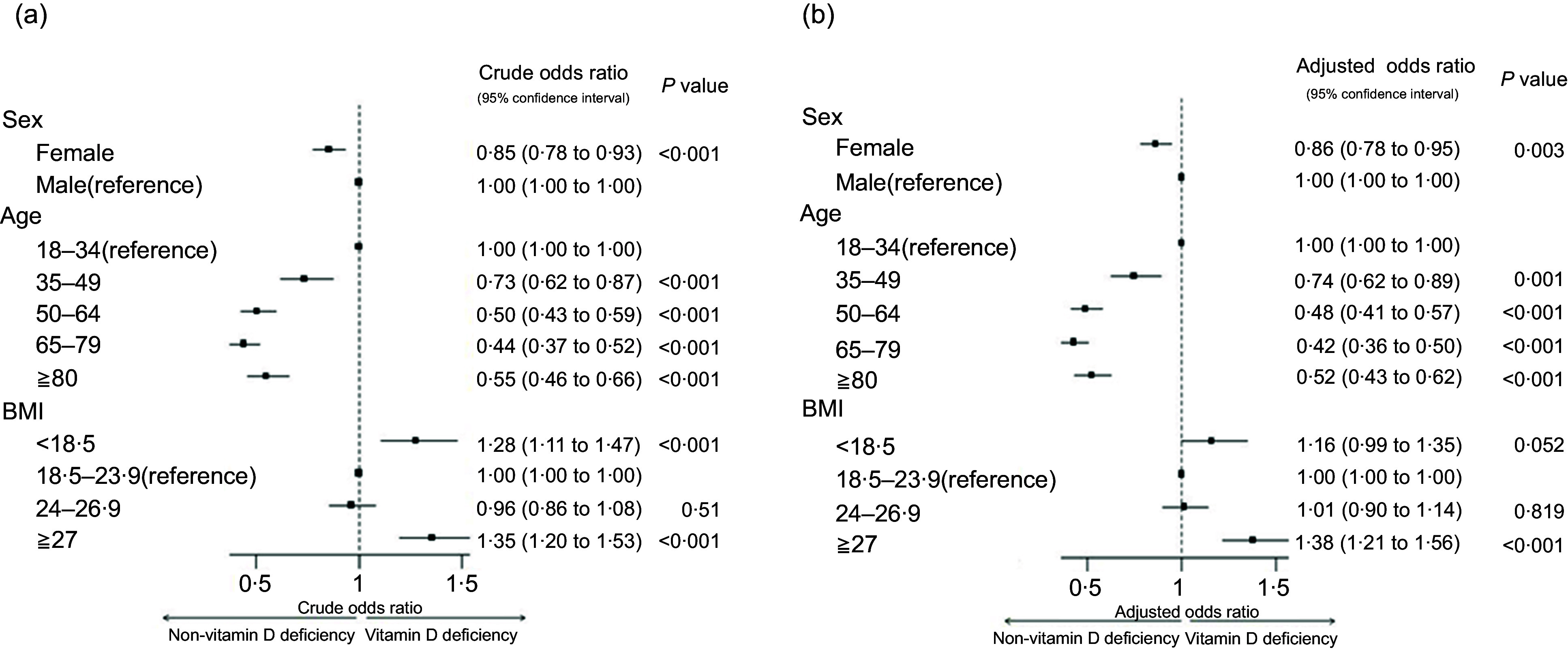
Forest plot: the multivariate analysis of the association between various variables and vitamin D deficiency in the study population (2013–2022). (a) Crude OR of vitamin D deficiency based on sex, age and BMI before adjustment for these variables. (b) Adjusted OR of vitamin D deficiency based on sex, age and BMI after adjustment for these variables.

The following prevalence and association analyses were based on serum vitamin D status at the time of each participant’s initial (first recorded) vitamin D test. Chi-square tests revealed significant differences in vitamin D deficiency prevalence across sex, age, BMI categories, medical specialties and comorbidities (Table 1). After adjustment, sex, age and BMI remained significantly associated with deficiency. Overall, 54·36 % of the study population was vitamin D deficient. Deficiency prevalence were higher in men (56·94 %) than in women (53·01 %), with an adjusted OR of 0·86 for women (95 % CI, CI: 0·78, 0·95, *P* = 0·003) (Figure 3). Vitamin D deficiency peaked in 2019–2020, with rates of 67·47 % in men and 63·85 % in women (Figure 2).

Younger adults (18–34 years) had the highest deficiency prevalence (67·81 %) and the lowest sufficiency prevalence (8·02 %) (Table 1). Participants aged 35 years or older had a lower risk of deficiency compared with those aged 18–34 years (Figure 3). Obesity was also significantly associated with higher deficiency prevalence (59·3 %), with an adjusted OR of 1·38 (95 % CI: 1·21, 1·56, *P* < 0·001) compared with individuals with normal BMI (Figure 3).

Significant differences in vitamin D deficiency prevalence were found across these various medical specialties and associated diseases or comorbidities. The highest deficiency prevalence, compared with the overall study population prevalence of 54·36 %, was noted in patients undergoing serum vitamin D testing in the chest medicine, OB/GYN and gastroenterology and hepatology departments (64·98 %, 63·28 % and 62·5 %, respectively). Additionally, patients with diagnosis of female infertility and endocrine disorders (except thyroid) exhibited the highest prevalence of deficiency (65·73 % and 62·67 %, respectively). Notably, in these specialties or related conditions, the vitamin D deficiency prevalence exceeded 60 %.

Compared with the nationally representative NAHSIT cohort (2017–2020), our study population had significantly lower mean serum 25-hydroxyvitamin D levels and a higher prevalence of vitamin D deficiency across most age and sex groups (see online supplementary material, Supplemental Table S3). For instance, among women aged 19–44 years, the deficiency prevalence in our cohort was 71·3 % compared with 42·9 % in NAHSIT.

### Changes in serum vitamin D status between initial and consecutive testing

**Figure 4.**
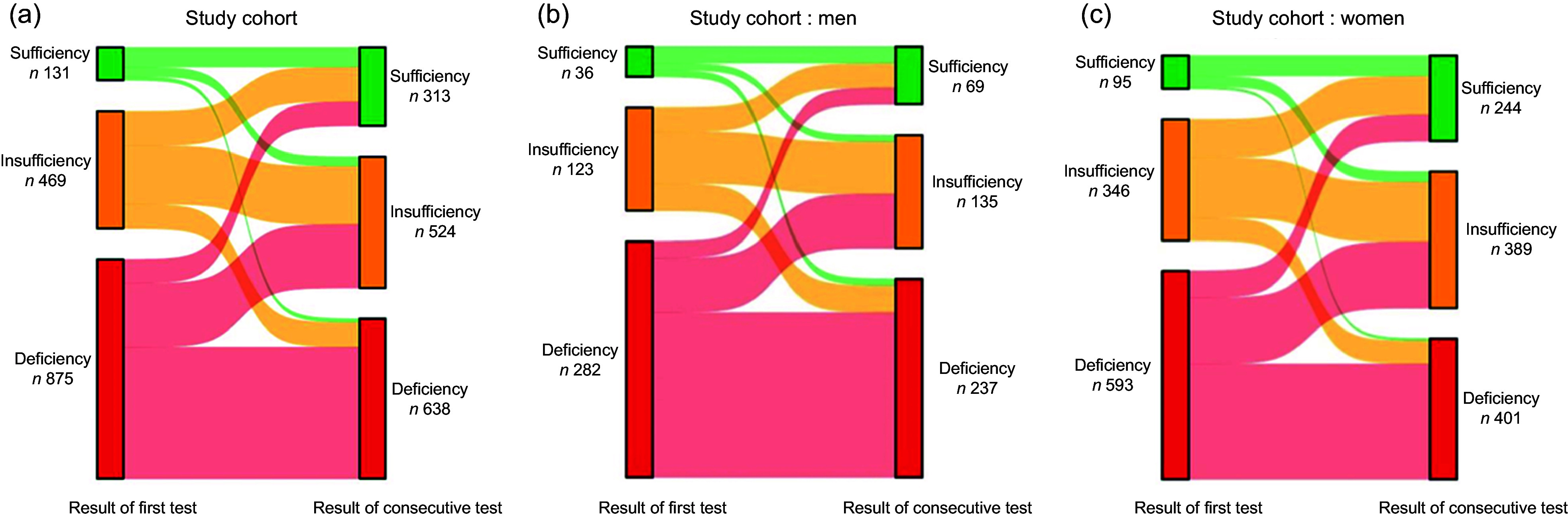
Sankey diagram of serum vitamin D status changes. The Sankey diagrams illustrate the dynamic changes in serum vitamin D status between the first and consecutive vitamin D testing for different cohorts within the study population. (a) All participants undergoing consecutive vitamin D testing. (b) Men undergoing consecutive vitamin D testing. (c) Women undergoing consecutive vitamin D testing.

Among the 1475 participants who underwent consecutive vitamin D tests within 2 years of their initial test (Table 2), dynamic changes in vitamin D status were observed. The interval between tests varied by baseline status, with shorter follow-up intervals among those initially deficient. At follow-up, deficiency prevalence declined to 43·25 %, and sufficiency increased to over 20 %. Two out of five initially deficient participants showed improvement. However, status deterioration was also observed: approximately 20 % of those initially insufficient and 40 % of those initially sufficient were reclassified into lower categories. These changes are illustrated in Figure 4.

**Table 2.** Characteristics of individuals with consecutive serum vitamin D testing by vitamin D status

Category	Total	Vitamin D status^*^ in the consecutive test					
		Deficiency		Insufficiency		Sufficiency	
	*n*	*n*	%	*n*	%	*n*	%
Individuals with consecutive tests	1475	638	43·25	524	35·53	313	21·22
Sex							
Male	441	237	53·74	135	30·61	69	15·65
Female	1034	401	38·78	389	37·62	244	23·60
Age years							
18–34	132	67	50·76	42	31·82	23	17·42
35–49	249	104	41·77	91	36·55	54	21·69
50–64	432	176	40·74	150	34·72	106	24·54
65–79	484	199	41·12	185	38·22	100	20·66
≥ 80	178	92	51·69	56	31·46	30	16·85
BMI^†^ (kg/m^2^) (58 missing data)							
Underweight	180	83	46·11	58	32·22	39	21·67
Normal	723	269	37·21	272	37·62	182	25·17
Overweight	296	125	42·23	113	38·18	58	19·60
Obese	218	155	71·10	78	35·78	33	15·14
Vitamin D status^*^ in the first test							
Deficiency	875	525	60·00	254	29·03	96	10·97
Insufficiency	469	98	20·90	232	49·47	139	29·64
Sufficiency	131	15	11·45	38	29·01	78	59·54

* Vitamin D status was defined as deficiency (< 20 ng/ml), insufficiency (20–29·9 ng/ml) and sufficiency (≥ 30 ng/ml).

† BMI categories are defined using the criteria of the Taiwanese Ministry of Health and Welfare: Underweight (BMI ≤ 18·5 kg/m^2^), normal weight (18·5 ≤ BMI < 24 kg/m^2^), overweight (24 ≤ BMI < 27 kg/m^2^) and obese (BMI ≥ 27 kg/m^2^) (Ministry of Health and Welfare in Taiwan. Evidence-based guideline on adult obesity prevention and management. Available at https://www.hpa.gov.tw/Pages/EBook.aspx?nodeid=1788 Accessed on December 22, 2023).

To assess whether the observed improvements in vitamin D status occurred beyond intra-individual variation, we recorded vitamin D status into a binary outcome (deficient *v*. non-deficient) and applied two statistical tests. A McNemar’s test comparing paired deficiency status at baseline and follow-up revealed a significant reduction in the number of individuals classified as deficient (*P* < 0·001), with more participants improving than worsening (350 *v*. 113). A paired *t* test comparing vitamin D levels before and after follow-up also showed a statistically significant increase (*P* < 0·001). These findings indicate that the overall improvement was unlikely to have occurred by chance.

## Discussion

Our study examined the trends and outcomes of voluntary vitamin D testing among outpatients in Taiwan over a decade (2013–2022). The findings reveal a notable rise in vitamin D testing, particularly after 2019, with higher utilisation among women (65·62 %) and older adults (70·14 % aged 50 years or older). Despite this, men and younger adults (18–34 years) exhibited higher deficiency prevalence, highlighting a discrepancy between test utilisation and actual deficiency prevalence. Additionally, a significant number of vitamin D tests were ordered by OB/GYN specialists, and individuals consulting an OB/GYN had some of the highest deficiency prevalence values, particularly those for conditions like infertility. These emphasise the importance of addressing vitamin D deficiency within specific demographics. Among the 1475 participants who underwent consecutive testing, the deficiency prevalence decreased from 60 % initially to 43·25 % at follow-up, with women demonstrating greater improvements than men.

Although our dataset did not specify whether vitamin D testing was initiated by physicians or requested by patients, the testing behaviours observed in our study likely reflect a combination of clinical judgement and individual health awareness. Notably, the prevalence of deficiency among individuals who underwent testing was markedly higher than that reported in the general population, as shown by comparisons with the nationally representative NAHSIT (2017–2020). This pattern, consistent across age and sex strata (see online supplementary material, Supplemental Table S3), suggests that the tested population may represent a higher-risk subgroup. These findings suggest that vitamin D testing in real-world clinical practice, though not systematically linked to documented indications, may therefore capture individuals at elevated risk who might not otherwise be identified through routine medical care.

### Utilisation patterns of vitamin D testing

Our findings show a marked increase in voluntary vitamin D testing, particularly after 2019. This trend aligns with the growing awareness of vitamin D’s role in health, which has been highlighted by numerous studies over the past two decades^(3–6)^. Increased public and scientific attention towards vitamin D has led to a surge in testing in various countries^(17–19)^. The COVID-19 pandemic further fueled this interest, as studies and media reports emphasise the potential role of vitamin D in immune support and its effects on COVID-19 outcomes^(28,29)^. This increased awareness likely contributed to the sharp rise in testing observed after 2019. Vitamin D deficiency peaked in 2019–2020, likely exacerbated by lifestyle changes during the pandemic. Lockdowns, remote work and reduced outdoor activities led to decreased sun exposure, resulting in lower vitamin D synthesis^(30,31)^. In the later stages of the pandemic, test utilisation remained high, but deficiency prevalence returned to pre-pandemic levels.

### Gender and age discrepancies in testing and deficiency

A notable finding in our study is the discrepancy between vitamin D testing utilisation and deficiency prevalence by gender. Although women were more likely to undergo testing, men exhibited higher prevalence of deficiency. This may be due to women’s greater health awareness and engagement in preventive health behaviours, as well as their higher utilisation of healthcare services compared with men^(32,33)^. These differences in health-seeking behaviour likely reflect broader gender-based patterns in medical care utilisation, contributing to more frequent vitamin D testing among women. Additionally, public health initiatives focusing on women’s bone health and reproductive care may further increase their interaction with healthcare providers and the likelihood of testing^(34)^. Women’s higher self-efficacy in managing health, such as adhering to supplementation and health advice, could also play a role^(35)^. In contrast, men had higher deficiency prevalence despite lower test uptake, potentially reflecting lower health awareness and greater reluctance to engage in preventive care^(36–38)^. These findings underscore the need for targeted public health strategies to improve screening and supplementation among men.

Younger adults (18–34 years) comprised a smaller proportion of those tested but exhibited the highest deficiency prevalence. Compared with older adults, younger individuals may perceive themselves at lower risk for chronic diseases, leading to reduced engagement in preventive behaviours such as supplementation, exercise or health screenings^(39)^. In contrast, studies show that older adults, driven by a higher awareness of their risk for chronic diseases, are more likely to participate in regular health screenings^(40,41)^. This likely contributes to the lower deficiency prevalence observed in older populations.

### Obesity and vitamin D deficiency

Obesity emerged as a significant factor associated with vitamin D deficiency. Vitamin D is fat-soluble and tends to be sequestered in adipose tissue, reducing its bioavailability in the bloodstream. Additionally, individuals with obesity may have lower levels of outdoor activity and sun exposure, further contributing to lower vitamin D synthesis^(42,43)^.

### Specialty and disease associations with vitamin D deficiency

Our study also shows the important role of OB/GYN in testing for vitamin D. The OB/GYN specialty accounted for the highest number of voluntary vitamin D tests, with about 30 % more tests ordered than endocrinology and nearly double the number ordered by nephrology. Notably, patients attending OB/GYN clinics also had some of the highest prevalence of vitamin D deficiency. While the exact clinical indications for vitamin D testing could not be directly determined from claims data, the associated diagnoses provide insight into the broader clinical and comorbidity context in which testing occurred. Additionally, female infertility was among the top disease groups associated with high deficiency prevalence. The intersection of high deficiency prevalence in the OB/GYN specialty and among patients for female infertility is particularly important. Previous research has shown that low vitamin D levels are linked to infertility, and vitamin D supplementation may improve reproductive outcomes in deficient individuals^(44–46)^. These findings indicate the importance of ensuring adequate vitamin D levels in women of reproductive age, particularly those seeking fertility treatment.

### Impact of consecutive testing on outcomes

Consecutive testing revealed significant improvements in vitamin D status over time, with the overall deficiency prevalence decreasing by 16·07 percentage points. The proportion of individuals with sufficient vitamin D levels more than doubled at follow-up. These findings suggest that vitamin D testing might be beneficial for managing nutrient deficiencies for specific high-risk groups. It offers timely opportunities for interventions to improve vitamin D levels.

The improvements observed align with the Health Belief Model, which posits that individuals are more likely to engage in preventive actions if they perceive a health threat and believe specific actions can mitigate it^(47,48)^. Informing patients of their deficiencies and suggesting management strategies or follow-up testing may encourage sustained health-promoting behaviours. However, not all participants improved, with some experiencing declines in vitamin D status. This dynamic change reflects the need for ongoing education and support to maintain adequate vitamin D levels^(49,50)^.

### Strengths and limitations

Our study has several strengths, including the use of a large sample size and a decade-long analysis. We minimised confounding by requiring a documented visit history of at least 2 years prior to the index date. This strengthened the validity of our longitudinal analysis. In addition, follow-up of consecutive vitamin D test levels enables us to evaluate the impact of the initial test on subsequent health outcomes, providing insights into the potential effectiveness of vitamin D testing for specific groups in clinical practice.

However, several limitations should be noted. As a retrospective cohort study, our reliance on outpatient diagnosis codes from medical records may not fully capture the clinical rationale or presenting symptoms that prompted vitamin D testing, which could affect the interpretability of disease associations. Our primary analysis used a one-year diagnostic window surrounding the index test to characterise broader comorbidity patterns, but this approach may have included conditions unrelated to the immediate decision to order testing. To address this, we conducted a sensitivity analysis using only same-day diagnoses from the index visit, which revealed substantial overlap with the 1-year data, particularly in endocrine, metabolic and gynaecologic conditions. These findings suggest that our results reflect stable comorbidity patterns across timeframes, though causality cannot be inferred.

Another limitation is the lack of recorded information on the specific vitamin D assay platform used. However, all tests were conducted at a single tertiary centre, which likely ensured consistent methodology and stable laboratory procedures over time, allowing for comparisons across years and subgroups within the study population. Nevertheless, caution is warranted when interpreting absolute vitamin D levels, given known variability – not only between assay types (e.g. immunoassays *v*. liquid chromatography–tandem MS (LC–MS/MS)) but also across laboratories using the same method^(51,52)^. These differences can influence clinical classification and should be considered when comparing results across studies.

Because vitamin D testing is self-paid in Taiwan, individuals from lower socio-economic backgrounds may be less likely to undergo testing, potentially contributing to disparities in access and detection. Economic considerations may also influence test-seeking behaviour across age groups: younger adults may be more cost sensitive or less motivated by preventive health concerns, while older adults may be more receptive to self-paid testing for health monitoring purposes. Additionally, individuals who voluntarily chose to undergo testing may have higher health awareness or preexisting health concerns, which could limit the generalisability of our results to the general population. However, our dataset did not include socio-economic or attitudinal variables, limiting our ability to assess how financial or behavioural factors influenced testing patterns. This limitation should be considered when interpreting our findings and planning future research.

In addition, our dataset did not allow us to determine whether vitamin D testing was initiated by patients or recommended by physicians. As testing is typically performed during outpatient visits and not reimbursed by insurance, the decision is likely shaped by both patient preferences and clinical judgment. These limitations should be considered when interpreting our findings and planning future research.

### Conclusion

This decade-long study demonstrated a marked increase in voluntary vitamin D testing among Taiwanese outpatients, especially after 2019, likely driven by heightened public awareness during the COVID-19 pandemic. Despite lower testing frequency, men and younger adults exhibited higher deficiency rates, underscoring demographic disparities and the importance of improved identification and monitoring strategies for at-risk populations. The observed improvements in vitamin D status among individuals undergoing consecutive testing further emphasise the potential public health benefits of personalised monitoring and tailored preventive strategies.

## Supporting information

Yeh et al. supplementary material Yeh et al. supplementary material

## Supplementary material

For supplementary material accompanying this paper visit https://doi.org/10.1017/S1368980025101183
