# Supplementary material for: Voluntary vitamin D testing: a decade-long study of utilisation patterns and impact on deficiency outcomes in Taiwan
Source: Public Health Nutr. 2025 Sep 25;28(1):e171. doi: 10.1017/S1368980025101183 (PMC12722091; doi:10.1017/S1368980025101183)
Supplement: Yeh et al. supplementary material [file S1368980025101183sup001.docx]

**Supplementary Material**

**Table S1** Medical Conditions Excluded from the Study Cohort

| Medical conditions | International Classification of Diseases, Clinical Modification, version 10 (ICD-10-CM) | International Classification of Diseases, Clinical Modification, version 9 (ICD-9-CM) |
| --- | --- | --- |
| Advanced renal failure or end-stage renal disease | N17, N18.6, N18.9, N19, N28.9, R39.2, R39.2, P96.0 | 585.4-585.6 |
| Rickets | E55.0, E64.3, E83.31, E83.32, K50, K90.0, N25.0 | 268.0, 268.2, 268.9, 579.0, 588.0 |
| Osteomalacia | M83.9 | 268.2 |
| Hyperparathyroidism | E21.0 | 252.0 |

**Table S2** Descriptive characteristics of the study population undergoing serum vitamin D testing (2013-2022)

| **Category** |  | **Year-period** | | | | |
| --- | --- | --- | --- | --- | --- | --- |
|  | Total  N(%) | 2013-2014  n(%) | 2015-2016  n(%) | 2017-2018  n(%) | 2019-2020  n(%) | 2021-2022  n(%) |
| **Overall** | 8383 | 447 | 1033 | 1292 | 2826 | 2785 |
| **Sex** | | | | | | |
| Male | 2882(34.38) | 190(42.51) | 532(51.50%) | 510(39.47) | 876(31.00) | 774(27.79) |
| Female | 5501(65.62) | 257(57.49) | 501(48.50%) | 782(60.53) | 1950(69.00) | 2011(72.21) |
| **Age (years)** | | | | | | |
| 18-34 | 873(10.41) | 16(3.58) | 107(10.36) | 141(10.91) | 275(9.73) | 334(11.99) |
| 35-49 | 1630(19.44) | 47(10.51) | 103( 9.97) | 198(15.33) | 586(20.74) | 696(24.99) |
| 50-64 | 2196(26.20) | 126(28.19) | 225(21.78) | 359(27.79) | 762(26.96) | 724(26.00) |
| 65-79 | 2397(28.59) | 140(31.32) | 313(30.30) | 395(30.57) | 837(29.62) | 712(25.57) |
| ≥80 | 1287(15.35) | 118(26.40) | 285(27.59) | 199(15.40) | 366(12.95) | 319(11.45) |

**Table S3** Comparison of serum 25(OH)D levels and vitamin D deficiency prevalence between the study and the NAHSIT population by sex and age group (2017–2020)

| Sex and age group | | Study population (2017–2020) | | | NAHSIT* population (2017–2020) | |
| --- | --- | --- | --- | --- | --- | --- |
|  |  | N tested | Mean 25(OH)D (ng/mL)  (SE) | Deficiency % (n/N) | Mean 25(OH)D (ng/mL) (SE) | Deficiency % |
| Men | 19-44 | 291 | 18.09(7.77) | 60.9% (187/291) | 28.5(0.51) | 14.9% |
|  | 45-64 | 437 | 17.50(8.05) | 65.7%(287/437) | 33.4(0.68) | 13.2% |
|  | ≥65 | 658 | 18.70(9.16) | 59.1%(389/658) | 37.6(0.61) | 5.4% |
| Women | 19-44 | 668 | 17.12(8.19) | 71.3%(476/668) | 22.1(0.42) | 42.9% |
|  | 45-64 | 925 | 19.81(10.10) | 56.6%(524/925) | 27.6(0.49) | 20.5% |
|  | ≥65 | 1139 | 19.42(8.83) | 57.5%(655/1139) | 31.6(0.55) | 13.5% |
| Total | 19-44 | 959 | 17.12(8.19) | 69.1%(663/959) | 25.4(0.37) | 28.7% |
|  | 45-64 | 1362 | 19.81(10.10) | 59.5%(811/1362) | 30.5(0.44) | 16.9% |
|  | ≥65 | 1797 | 19.42(8.83) | 58.1%(1044/1797) | 34.3(0.50) | 9.8% |

***Note:**
This table presents a stratified comparison of vitamin D status between individuals undergoing first-time vitamin D testing at our medical center and participants in the Nutrition and Health Survey in Taiwan (NAHSIT), both during 2017–2020. NAHSIT is a nationally representative survey conducted by the Health Promotion Administration using a multistage stratified probability sampling design. For comparability, our study cohort was restricted to individuals who received their first vitamin D test during the same period, and data were stratified by sex and age groups consistent with the NAHSIT classification.

Vitamin D deficiency was defined as serum 25-hydroxyvitamin D (25(OH)D) <20 ng/mL, and insufficiency as 20–29.9 ng/mL.

**Abbreviations:** NAHSIT, Nutrition and Health Survey in Taiwan; 25(OH)D, 25-hydroxyvitamin D; VitD, vitamin D; SE, standard error.

**Source:**
Nutrition and Health Survey in Taiwan (NAHSIT), 2017–2020. Health Promotion Administration, Ministry of Health and Welfare, Taiwan.
Available at: <https://www.hpa.gov.tw/EngPages/Detail.aspx?nodeid=3999&pid=15562> (accessed July 5, 2025).
Survey description: <https://www.hpa.gov.tw/EngPages/List.aspx?nodeid=3998>

**Table S4** Common diagnostic categories at the index visit, stratified by vitamin D status and classified using Clinical Classifications Software (CCS).

| CCS Category (Diagnosis Group) | Deficient (n) | Insufficient (n) | Sufficient (n) |
| --- | --- | --- | --- |
| Diseases of female genital organs | 879 | 471 | 122 |
| Other endocrine disorders | 475 | 204 | 51 |
| Thyroid disorders | 339 | 194 | 63 |
| Hypertension | 326 | 224 | 83 |
| Diseases of the urinary system | 309 | 206 | 90 |
| Upper gastrointestinal disorders | 285 | 185 | 73 |
| Diseases of the heart | 276 | 173 | 48 |
| Other gastrointestinal cancer | 276 | 94 | 29 |
| Diabetes mellitus without complication | 275 | 171 | 48 |
| Symptoms; signs; and ill-defined conditions | 248 | 87 | 34 |
| Viral infection | 234 | 162 | 52 |
| Disorders of lipid metabolism | 227 | 187 | 78 |
| Benign neoplasms | 218 | 148 | 56 |
| Eye disorders | 199 | 116 | 77 |
| Osteoporosis | 169 | 189 | 107 |

Note: Diagnoses were recorded from the same ambulatory visit on which the index vitamin D test was ordered. Frequencies are presented separately for individuals classified as vitamin D deficient, insufficient, or sufficient.

Abbreviations: CCS, Clinical Classifications Software; VitD, vitamin D.
